# Tetracycline reprograms inflammatory and regenerative signaling pathways in human keratinocytes exposed to *Loxosceles* spider venoms and sphingomyelinases

**DOI:** 10.3389/fphar.2026.1783681

**Published:** 2026-04-22

**Authors:** Bruna Fernandes Pinto, Priscila Hess Lopes, Carlos Eduardo Madureira Trufen, Ana Tung Ching Ching, Inácio de Loiola Meirelles Junqueira de Azevedo, Milton Yutaka Nishiyama-Jr, Denise V. Tambourgi

**Affiliations:** 1 Immunochemistry Laboratory, Butantan Institute, São Paulo, Brazil; 2 PREVOR, Liège, Belgium; 3 Laboratory of Applied Toxinology, Butantan Institute, São Paulo, Brazil

**Keywords:** *Loxosceles* venom, sphingomyelinase D, tetracycline, wound healing, cutaneous loxoscelism treatment

## Abstract

**Introduction:**

*Loxosceles* spider envenomation, or loxoscelism, constitutes the most severe form of araneism and frequently progresses to dermonecrosis with significant tissue damage. The key venom component, sphingomyelinase D (SMase D), drives both local and systemic effects through its structurally distinct Class I and II isoforms, each differing in toxicity. The current therapies provide limited benefit and, once necrosis is established, interventions are primarily supportive, underscoring the need for more effective pharmacological options. While tetracyclines have emerged as promising modulators of cutaneous loxoscelism in animal models, beyond their antimicrobial properties and owing to their ability to inhibit matrix metalloproteinases, the molecular mechanisms underlying their protective effects remain poorly defined.

**Methods:**

This study aimed to elucidate the transcriptomic landscape of tetracycline-associated protection in human keratinocytes in response to *Loxosceles* venoms and SMase D Class I and II isoforms.

**Results:**

Using transcriptomic profiling, we show that tetracycline upregulates *SOX2* and *SOX18* while downregulating IL1RL1 in keratinocytes exposed to *Loxosceles* venoms and SMases D. These regulatory changes are associated with reduced IL-1-mediated inflammation and activate pathways related to cell migration, epidermal morphogenesis, and tissue regeneration. Gene Ontology enrichment supported these findings, linking tetracycline treatment to biological processes of proliferation, wound closure, and repair. Furthermore, tetracycline attenuates SMase D-induced expression of pro-inflammatory and proteolytic mediators, shifting gene expression patterns toward profiles compatible with tissue homeostasis.

**Conclusion:**

Collectively, these transcriptomic findings, together with our previous functional studies, support a mechanistic framework in which tetracycline mitigates venom-induced pathology and highlight its potential as a therapeutic candidate for cutaneous loxoscelism and warrants targeted functional validation in future studies.

## 1 Introduction

Envenomation by spiders of the genus *Loxosceles*, termed loxoscelism, constitutes the most clinically severe form of araneism (Lopes et al., 2020). Epidemiological surveillance data from Brazil recorded more than 9,000 cases in 2024 (Sistema de Informação de Agravos de Notificação Brasil, 2025). Although systemic manifestations such as hemolysis, disseminated intravascular coagulation, and acute renal failure can occur, the cutaneous form accounts for most of the clinical burden because local lesions are highly prevalent (Futrell, 1992; Lopes et al., 2020).

The hallmark of loxoscelism, cutaneous necrosis, expands progressively, destroys tissue, and heals slowly, sometimes requiring months for complete re-epithelialization. This severe pathology results from venom components that trigger synergistic inflammatory and proteolytic cascades (Wasserman and Anderson, 1983; Tambourgi et al., 2010).

The principal toxin in *Loxosceles* venoms, Sphingomyelinase D (SMase D), is firmly established as the critical mediator of both systemic and local tissue damage that characterize the clinical variants of loxoscelism (Tambourgi et al., 1995; Tambourgi et al., 1998; Tambourgi et al., 2000; Tambourgi et al., 2002; Tambourgi et al., 2004; Tambourgi et al., 2005; Tambourgi et al., 2007).

Mechanistically, SMase D remodels the architecture of plasma membrane lipid rafts, triggering the activation of ADAM metalloproteinases and subsequent cell-surface proteins shedding (van den Berg et al., 2002; 2007; 2012; Lopes et al., 2020b). This signaling sustains persistent neutrophil infiltration and induces matrix metalloproteinases (MMP) expression. Together, these processes drive substantial proteolytic degradation and the dissociation of dermal collagen networks, thereby fueling the development of necrotic ulcerations (Tambourgi et al., 2005; Paixao-Cavalcante et al., 2006; Tambourgi et al., 2010; Corrêa et al., 2016).

Structurally, SMase D isoforms are classified into two distinct classes: Class I isoforms, exemplified by *Loxosceles laeta*, which contain a single disulfide bridge, and Class II isoforms, typical of *Loxosceles intermedia*, distinguished by an additional disulfide bond linking the flexible and catalytic loops (Fernandes Pedrosa et al., 2002; Tambourgi et al., 2004; Zela et al., 2004; Murakami et al., 2005; 2006; Pedroso et al., 2015). Consistent with this structural divergence, previous results from our group demonstrate that *L. laeta* venom and Class I SMase D exhibit greater dermonecrotic activity than *L. intermedia* venom and Class II SMase D, underscoring a species- and isoform-dependent toxicity that challenges clinical management (de Oliveira et al., 2005; de Santi Ferrara et al., 2009; Corrêa et al., 2016; Pinto et al., 2025).

Therapeutic strategies for loxoscelism remain largely unsatisfactory, with successful outcomes depending on the timing and progression of the lesion (Malaque et al., 2022). Diverse approaches, ranging from antivenom, systemic corticosteroids, dapsone, antihistamines, antibiotics, analgesics, and hyperbaric oxygen therapy, to surgical interventions such as debridement and grafting, have been investigated, but the clinical outcomes remain suboptimal, and may entail unwanted adverse effects. Moreover, diagnosis is complicated by delayed patient presentation and the lack of specific laboratory tests (Isbister and Fan, 2011; Rees et al., 1981; Lopes et al., 2020). These limitations underscore the urgent need for therapies that directly target the venom-induced proteolytic cascades driving dermonecrosis.

Given the proposed role of metalloproteinases in dermonecrosis development, therapeutic agents targeting these enzymes, particularly tissue inhibitors of metalloproteinases (TIMPs) and tetracyclines, have emerged as promising therapeutic candidates (Pasternak and Aspenberg, 2009). In addition to the well-recognized antimicrobial effects, tetracyclines inhibit MMPs by chelating zinc, blocking pro-MMP maturation, and downregulating MMP genes expression (Acharya et al., 2004). However, the mechanisms by which tetracycline modulates human keratinocyte signaling pathways during venom exposure remain unclear.

In experimental models of loxoscelism, tetracyclines reduce the secretion and activity of MMP-2 and MMP-9, and dampen the activation of pro-MMP-7 by *L. laeta* venom. Consistently, topical application of tetracycline cream promote healing of venom-induced dermonecrosis in rabbits (Paixao-Cavalcante et al., 2006; Paixao-Cavalcante et al., 2007; Corrêa et al., 2016). Despite these promising effects, the precise intracellular signaling pathways modulated by tetracycline in human skin cells exposed to *Loxosceles* venom remain largely unclear. We therefore investigated these molecular actions of tetracycline in human keratinocytes, the predominant type of skin cells, exposed to whole venoms and SMase D isoforms from *L. laeta* and *L. intermedia*.

## 2 Materials and methods

### 2.1 *Loxosceles* spider venom

Adult female *L. laeta* and *L. intermedia* spiders were supplied by the Immunochemistry Laboratory at the Butantan Institute, São Paulo, Brazil, under IBAMA capture and maintenance license 45166–6. Venom extraction was performed by electrostimulation following the protocol originally described by Bucherl (1969) with minor adaptations. Briefly, electrical pulses (15–20 V) were repeatedly applied to the spider’s sternum, and the venom droplets were collected into sterile phosphate-buffered saline (PBS) using a micropipette. The protein concentrations of venoms were determined using the Bicinchoninic Acid (BCA) Protein Assay Kit (Pierce Biotechnology, Inc., Waltham, MA, USA) according to the manufacturer’s instructions, and venoms were then aliquoted and stored at −20 °C until use. Authorization for access to genetic resources was granted under process number 02001.008541/2011–52, authorization 01/2009.

### 2.2 Recombinant sphingomyelinase D production

Recombinant sphingomyelinase D (SMase D) isoforms from *L. laeta* and *L. intermedia* venoms were cloned, expressed, and purified as previously described by Fernandes Pedrosa et al. (2002) and Tambourgi et al. (2004), respectively. Protein concentrations were quantified using the Lowry assay (Lowry et al., 1951). The purity of the recombinant proteins was assessed by 12% SDS-PAGE under non-reducing conditions (Laemmli, 1970). Authorization for genetic resources was granted by the National System for the Management of Genetic Heritage and Associated Traditional Knowledge (SisGen; registration number AEE9AEA, 11/01/2018).

### 2.3 Cell culture

The human keratinocyte cell line HaCaT (Banco de Células do Rio de Janeiro, BCRJ, Brazil) was cultured in 75 cm^2^ flasks (Corning Inc., New York, NY, USA) in Dulbecco’s Modified Eagle Medium (DMEM) supplemented with 10% fetal bovine serum (FBS) and 1% penicillin-streptomycin. Cultures were maintained at 37 °C in a humidified incubator with 5% CO_2_.

### 2.4 RNA-seq analysis

#### 2.4.1 Cell treatments and data collection

HaCaT keratinocyte cells were incubated overnight in serum-free DMEM. Cells were then detached using ATV solution (0.2% trypsin and 0.02% Versene), washed, and resuspended in serum-free DMEM. Triplicate aliquots of the cell suspension (1 × 10^6^ cells/mL) were incubated under gentle agitation for 2 h at 37 °C in serum-free DMEM supplemented with VBS^++^ buffer (2.8 mM barbituric acid, 145.5 mM NaCl, 0.8 mM MgCl_2_, 0.3 mM CaCl_2_, 0.9 mM sodium barbital; pH 7.2) and treated with either *L. laeta* or *L. intermedia* venom (10 μg/mL), or with recombinant Class I or Class II SMases D (5 µg final concentration), with or without tetracycline (50 μg/mL). PBS with or without tetracycline were used as controls. After incubation, cells were washed with serum-free DMEM and centrifuged at 1,500 rpm for 10 min at 4 °C before RNA extraction. Total RNA extraction and library preparation were performed as described in Pinto et al. (2023).

#### 2.4.2 Sequencing

cDNA libraries were pooled at a final concentration of 12 pM and sequenced on an Illumina HiSeq 1,500 System (Illumina, San Diego, CA, EUA) in Rapid Run mode, using paired-end 200 cycles of 2 * 101 bp chemistry. Raw data preprocessing, quality filtering, genome mapping, transcript assembly, and quantification followed the workflow as previously described (Pinto et al., 2023).

#### 2.4.3 Differential gene expression and functional analyses

Read counts were normalized using the RUVg function from the RUVSeq package to control for batch effects (Risso et al., 2014). Differential gene expression analysis was assessed with the edgeR package (Robinson et al., 2010). Normalization factors were calculated using the trimmed mean of M values (TMM) method (Robinson and Oshlack, 2010). Dispersion estimates were obtained using the estimateDisp function (Chen et al., 2014; Phipson et al., 2016), and gene-wise negative binomial generalized linear models were fit using glmFit (McCarthy et al., 2012). Genes were defined as differentially expressed (DEGs) when exhibiting an absolute log_2_ fold change greater than one and a false discovery rate (FDR)-adjusted p-value less than 0.05 (Benjamini and Hochberg, 1995). Volcano plots were generated in GraphPad Prism 8.0 to visualize these DEGs.

Gene Ontology (GO) enrichment analysis for biological processes was performed as described by Harris et al. (2004). Protein-protein interaction networks were constructed using STRING (Szklarczyk et al., 2021) and visualized in Cytoscape (Shannon et al., 2003). Pathway enrichment analysis was performed using the Reactome database via EnrichR (Kuleshov et al., 2016), applying a significance threshold of -log_10_ Adjusted p-value greater than 1.3.

## 3 Results

### 3.1 Tetracycline orchestrates opposing regulation of *SOX2/SOX18* and *IL1RL1* in keratinocytes challenged with *Loxosceles* venoms or SMases D

**FIGURE 1:**
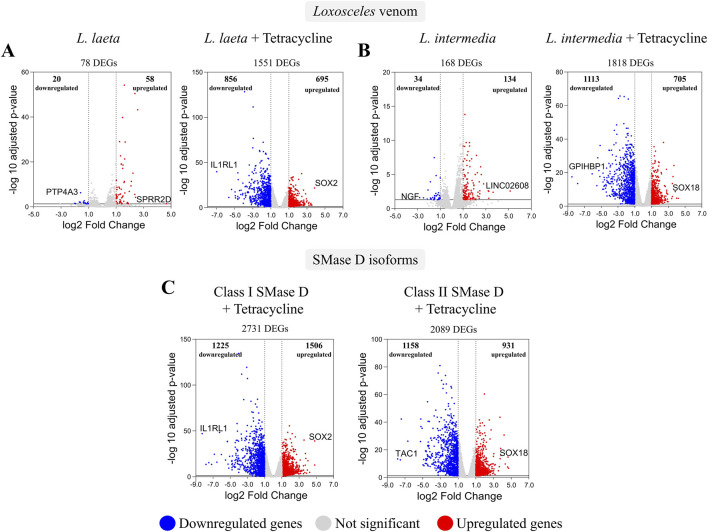
Differential gene expression in keratinocytes exposed to *Loxosceles* venoms and recombinant SMases D, with or without tetracycline. Volcano plots showing differentially expressed genes (DEGs) after 2 h of treatment with **(A)** *L. laeta* venom or **(B)** *L. intermedia* venom, in the absence or presence of tetracycline. **(C)** Volcano plot depicting DEGs after 2 h of treatment with Class I and Class II SMases D with tetracycline. The most upregulated genes are shown in red, the most downregulated genes in blue, and non-significant genes in grey. Genes were considered differentially expressed when log2 fold change (log2FC) ≥ 1 or ≤ −1 with FDR <0.05.

Transcriptomic analysis revealed that *Loxosceles* venoms induce distinct gene expression profiles in human keratinocytes, which are markedly modulated by tetracycline co-treatment. Treatment with *L. laeta* venom alone induced 78 differentially expressed genes (20 downregulated, 58 upregulated), whereas *L. intermedia* venom induced 168 (34 downregulated, 134 upregulated), relative to PBS-treated control cells. Notably, co-treatment with tetracycline markedly increased the number of DEGs, yielding 1,551 (856 downregulated, 695 upregulated) for *L. laeta* venom and 1,818 (1,113 downregulated, 705 upregulated) for *L. intermedia* venom, both relative to PBS plus tetracycline as control*.* In particular, the angiogenesis-, cell migration- and regeneration-associated transcription factors, *SOX2* and *SOX18,* emerged as the most robustly induced genes in the presence of venom plus tetracycline, whereas the pivotal mediator of several inflammatory cascades, *IL1RL1*, was the most consistently repressed (Figures 1A,B).

A similar pattern emerged when keratinocytes were treated with recombinant SMase D. Co-treatment with Class I and Class II SMase D isoforms plus tetracycline induced 2,731 DEGs (1,225 downregulated, 1,506 upregulated) and 2,089 DEGs (1,158 downregulated, 931 upregulated), respectively. Consistent with previous observations, *SOX2* and *SOX18* were among the most strongly upregulated genes, while *IL1RL1* showed the most pronounced downregulation following tetracycline co-treatment (Figure 1C). Collectively, these findings reveal that tetracycline mediates coordinated transcriptional remodeling in keratinocytes exposed to either *Loxosceles* venoms or SMase D isoforms, potentiating regenerative pathways while suppressing key inflammatory drivers.

### 3.2 Tetracycline restores wound healing pathways disrupted by *Loxosceles* venoms

**FIGURE 2:**
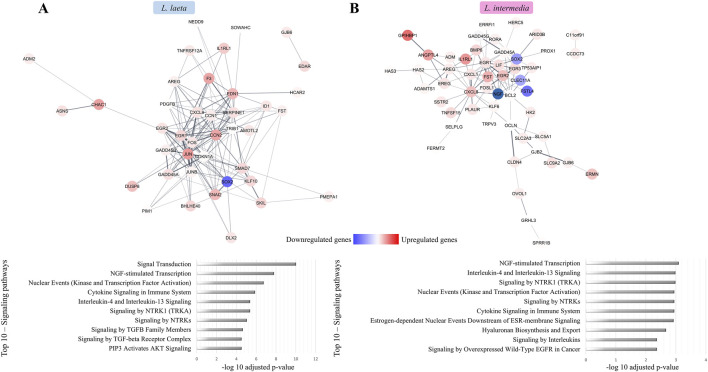
Network analysis of differentially expressed genes following *Loxosceles* venoms treatment. Interaction networks of DEGs identified in keratinocytes exposed to **(A)** *L. laeta* and **(B)** *L. intermedia* venoms were constructed using STRING v12.0 and visualized in Cytoscape v3.10.3. The networks are annotated with the top 10 enriched signaling pathways associated with each treatment, identified using the EnrichR platform (Reactome database; FDR-adjusted enrichment scores).

Interaction network analysis of DEGs in keratinocytes exposed to whole *L. laeta* venom revealed a dense cluster of upregulated genes involved in inflammation, apoptosis, and coagulation cascade activation, alongside suppression of the reparative factor *SOX2* (Figure 2A). Among the top 10 signaling categories identified in the interaction network, signal transduction, cytokine signaling within the immune system, interleukin-4 and interleukin-13 signaling, and pathways mediated by the TGF-β family and TGF-β receptor complex were strongly represented (Figure 2A). Likewise, network analysis of DEGs triggered by *L. intermedia* venom demonstrated a predominance of pro-inflammatory gene networks and reduced expression of cell proliferation-associated pathways, a mixture of pro- and anti-apoptotic activities, and impaired migratory behavior essential for epidermal repair (Figure 2B). In this context, downregulated genes were primarily associated with reduced proliferative capacity of keratinocytes. Top 10 enriched networks emphasized the importance of NGF-stimulated transcription, interleukin-4 and interleukin-13 signaling, general cytokine signaling, and, notably, signaling by overexpressed wild-type EGFR, a key axis in wound healing and epithelial regeneration (Figure 2B).

**FIGURE 3:**
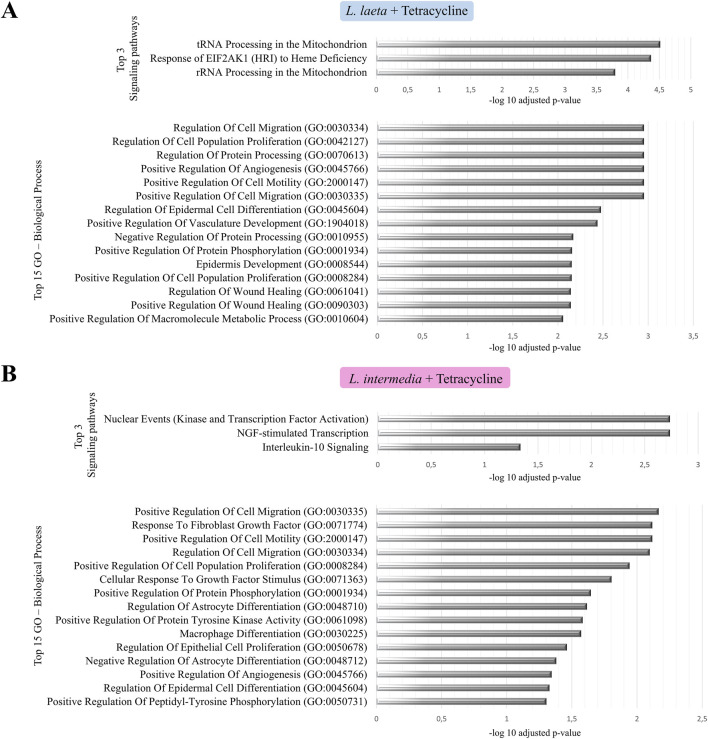
Functional enrichment analysis of differentially expressed genes in keratinocytes treated with *Loxosceles* venoms and tetracycline. Top 3 enriched signaling pathways and top 15 enriched biological processes are shown for keratinocytes treated with **(A)** *L. laeta* venom plus tetracycline and **(B)** *L. intermedia* venom plus tetracycline. Biological processes were analyzed using the Gene Ontology (GO) database, and pathways were identified using the Reactome database via EnrichR. Enrichments with −log10(FDR) > 1.3 were considered significant.

In contrast, tetracycline co-treatment significantly reprogrammed these venom-driven profiles. For *L. laeta* venom, signaling pathway enrichment analysis highlighted tRNA and rRNA mitochondrial processing as dominant pathways, suggesting restoration of protein synthesis machinery that may support tissue repair. GO analysis of the 15 most significantly enriched biological processes showed an increased representation of pathways related to cell migration and proliferation, angiogenesis, epidermal differentiation, and multiple dimensions of wound healing (Figure 3A).

Similarly, for *L. intermedia* venom, in the presence of tetracycline, interleukin-10 signaling, a central anti-inflammatory axis, emerged as a principal pathway. GO enrichment pinpointed robust activation of processes critical for regenerative outcomes, including cell migration, response to fibroblast growth factors, epithelial and vascular proliferation, and epidermal differentiation (Figure 3B). Together, these data demonstrate that disruption of wound healing pathways induced by *Loxoscele*s venoms is reversed by tetracycline, which not only dampens inflammatory and apoptotic gene signatures but also reinstates transcriptional programs toward gene networks associated with regenerative and differentiation processes.

### 3.3 Tetracycline modulates SMase D-induced inflammatory and proteolytic gene expression in keratinocytes

**FIGURE 4:**
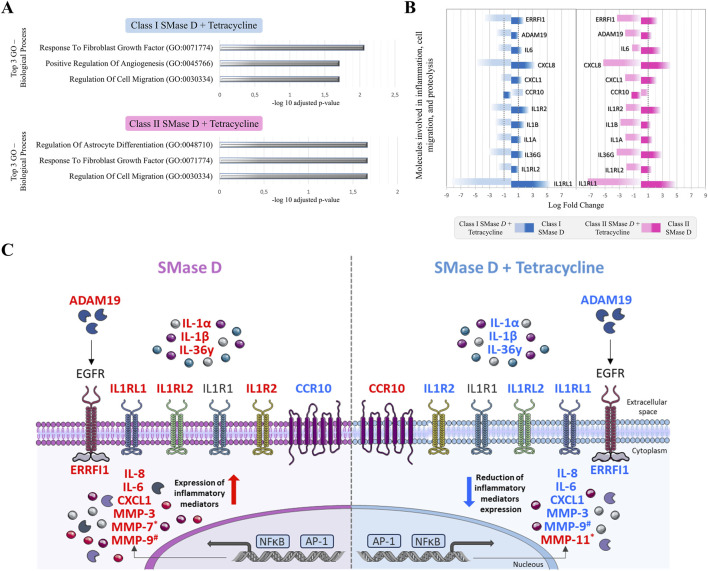
Functional enrichment and signaling pathways regulated by recombinant SMase D in the presence of tetracycline. **(A)** Top 3 biological processes enriched in keratinocytes treated with Class I or Class II SMase D plus tetracycline. **(B)** Log2 fold change values of key inflammatory and proteolytic molecules after treatment with Class I and Class II SMase D, with or without tetracycline. **(C)** Schematic representation of molecules and signaling pathways involved in SMase D-induced inflammatory responses and impaired wound healing, and their modulation by tetracycline. Molecules shown in red are upregulated and those in blue are downregulated according to the transcriptomic analysis. Asterisks (*) indicate significant differences relative to Class I SMase D alone, and hash symbols (#) indicate significant differences relative to Class II SMase D alone (FDR <0.05). The scheme was adapted from Servier Medical Art (https://smart.servier.com), licensed under CC BY 4.0 (https://creativecommons.org/licenses/by/4.0/).

In recent work from our group, we demonstrated that exposure of keratinocytes to SMase D strongly activates gene networks associated with inflammatory signaling and significantly impairs the wound-healing process (Pinto et al., 2023; 2025). In the present study, transcriptomic analysis in the context of tetracycline co-treatment revealed a markedly different gene expression landscape. GO analysis of cells co-treated with tetracycline revealed “response to fibroblast growth factor” and “regulation of cell migration” as the most prominently enriched biological processes after exposure to either Class I or Class II SMases D (Figure 4A).

Notably, tetracycline treatment reversed the upregulation of inflammatory and proteolytic genes previously observed under Class I and Class II SMase D exposure (Pinto et al., 2023), *ERRFI1*, *ADAM19*, *IL6*, *CXCL8*, *CXCL1*, *IL1R2*, *IL1B*, *IL1A*, *IL36G*, *IL1RL2* and *IL1RL1*. In addition, tetracycline also upregulated *CCR10*, a gene linked to reduced inflammatory mediator expression and epithelial homeostasis (Figures 4B,C).

**FIGURE 5:**
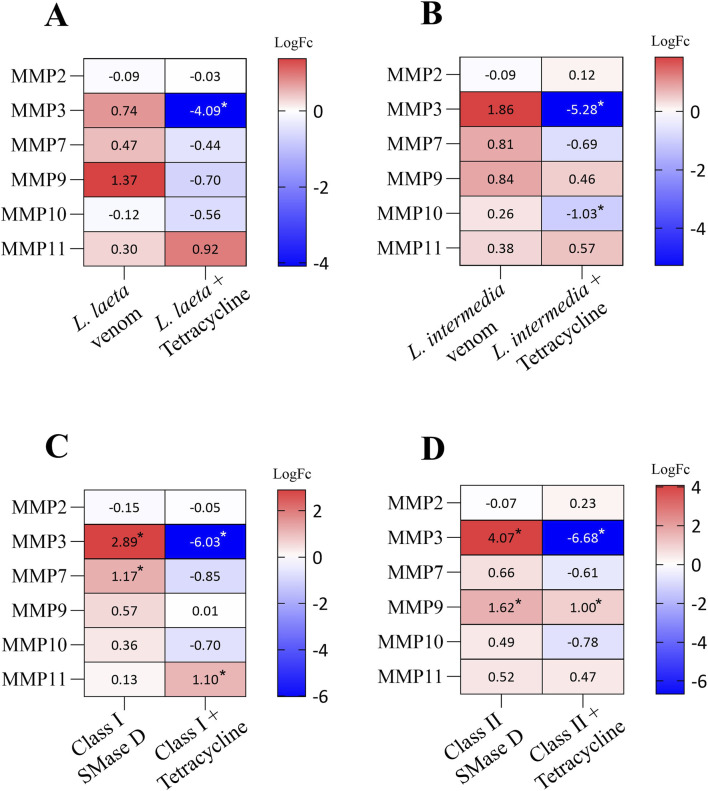
Differential expression of matrix metalloproteinase (MMP) genes in keratinocytes exposed to *Loxosceles* venoms and SMases D isoforms, with or without tetracycline treatment. **(A,B)** Log2 fold change (log2FC) values for MMP transcripts after exposure to *L. laeta* and *L. intermedia* venoms, respectively, in the absence or presence of tetracycline. **(C,D)** Log2FC values for MMP transcripts after treatment with Class I and Class II SMase D, respectively, with or without tetracycline. Tetracycline was evaluated as a modulator of venom- and toxin-induced transcriptional responses. Heatmaps were generated using GraphPad Prism v10.6.1. Color gradients indicate the magnitude and direction of gene expression changes (red: upregulation; blue: downregulation). Genes were considered differentially expressed when log2FC ≥ 1 or ≤ −1 with FDR <0.05 and are marked with an asterisk (*).

In the context of MMP regulation, tetracycline markedly reduced *MMP3* expression in *L. laeta* venom-treated cells (LogFC = −4.09; adjusted p = 2.26 × 10^−16^), as well as *MMP3* (LogFC = −5.28; adjusted p = 5.48 × 10^−22^) and *MMP10* (LogFC = −1.03; adjusted p = 5.39 × 10^−20^) in *L. intermedia* venom-treated cells (Figures 5A,B). On the other hand, Class I SMase D treatment led to significant upregulation of *MMP7* gene (LogFC = 1.17; adjusted p = 1.04 × 10^−4^), as previously reported at the protein level (Corrêa et al., 2016), along with increased *MMP3* expression (LogFC = 2.89; adjusted p = 5.39 × 10^−5^). These effects were reversed by tetracycline, with *MMP7* returning to basal levels (LogFC = −0.85; adjusted p = 5.66 × 10^−3^) and *MMP3* showing pronounced downregulation (LogFC = −6.03; adjusted p = 4.29 × 10^−25^). In this same context, tetracycline increased *MMP11* expression (LogFC = 1.1; adjusted p = 5.73 × 10^−03^) (Figure 5C). Finally, Class II SMase D treatment induced *MMP3* (LogFC = 4.07; adjusted p = 1.29 × 10^−10^) and *MMP9* expression (LogFC = 1.62; adjusted p = 9.17 × 10^−04^) and tetracycline substantially reduced both *MMP9* (LogFC 1.0; p = 1.46 × 10^−03^) and *MMP3* expression (LogFC = −6.68; adjusted p = 4.66 × 10^−27^) in this setting (Figure 5D).

## 4 Discussion

In this study, we performed a comprehensive RNA-seq analysis of human keratinocytes exposed to *L. laeta* and *L. intermedia* venoms and to recombinant SMase D Class I and II isoforms, with or without tetracycline co-treatment, to delineate the transcriptional programs associated with venom-induced damage and tetracycline-mediated protection. Rather than providing exhaustive functional validation, our goal was to generate an integrated transcriptomic and network-based mechanistic framework that extends our previously reported *in vitro* and *in vivo* functional studies in models of loxoscelism and tetracycline therapy.

Despite extensive research on loxoscelism, management of venom-induced dermonecrosis and systemic complications remains controversial. Different therapeutic approaches are required depending on the severity of the dermonecrotic lesion and the degree of progression to serious systemic complications. Therapeutic failure is frequently attributable to delayed medical care, since bites from *Loxosceles* spiders are initially painless and most patients remain unaware of the envenomation until 2–8 h post-bite, when moderate to severe pain and localized edema typically develop (Futrell, 1992; Hogan et al., 2004; Tambourgi et al., 2010; Lopes et al., 2020). Conventional therapies for cutaneous loxoscelism, such as corticosteroids, antibiotics, antihistamines, dapsone, hyperbaric oxygen, and antivenom, have demonstrated limited efficacy and, in some cases, undesirable adverse effects, as previously discussed. Concomitantly, diagnosis remains imprecise, since both systemic and particularly local manifestations are nonspecific and may resemble other conditions considered in the differential diagnosis of loxoscelism (Lopes et al., 2020). Thus, the identification of an effective therapy capable of preventing the progression of cutaneous lesions to necrotic conditions remains a major challenge. In this context, dissecting tetracycline-responsive pathways in keratinocytes may help to inform more targeted therapeutic strategies.

Considering the well-established antibiotic and anti-inflammatory properties of tetracycline, along with our previous experimental findings showing that tetracycline prevents lesion development both *in vitro* and *in vivo* models of loxoscelism, and also reduces kidney damage induced by *Loxosceles* spider venom (Paixao-Cavalcante et al., 2006; Paixao-Cavalcante et al., 2007; Corrêa et al., 2016; Okamoto et al., 2017), we sought to investigate its action at the molecular level. Thus, our study focuses on the intracellular responses of human keratinocytes exposed to *Loxosceles* venoms or its main toxin, SMase D, with or without tetracycline treatment.

Keratinocytes exposed to venoms from *L. laeta* and *L. intermedia* displayed differentially expressed genes (DEGs) associated with inflammatory activation, pro- and anti-apoptotic pathways, coagulation and thrombo-inflammation, proteolytic activity, impaired cell migration, and defective wound healing (notably *ERRFI1* upregulation and *SOX*2 downregulation). ERRFI1 functions as a potent inhibitor of epidermal growth factor receptor (EGFR), binding to its kinase domain and stabilizing the receptor in an inactive conformation (Zhang et al., 2007; Cairns et al., 2018). Since EGFR signaling orchestrates cutaneous wound repair, the combined effect of ERRFI1-mediated kinase inhibition and SMases D-driven ADAM-dependent EGFR cleavage, disrupt receptor function, explaining the impaired re-epithelialization observed in clinical and experimental loxoscelism (van den Berg et al., 2002; Tambourgi et al., 2010; Lopes et al., 2020b; Pinto et al., 2023). In addition, ERRFI1 has been implicated in promoting apoptosis and inhibiting cell proliferation in several cancer models (Lin et al., 2011; Cui et al., 2021), also consistent with our previous findings that SMase D from *Loxosceles* venom triggers keratinocyte apoptosis, characterized by loss of membrane asymmetry and DNA fragmentation (Tambourgi et al., 2002; Paixao-Cavalcante et al., 2006). Altogether, these data indicate that, in line with studies employing SMase D, whole crude venoms also provoke an inflammatory gene-expression program and activate proteolytic cascades that culminate in tissue destruction.

It is important to note that methodological differences between whole-venom and recombinant SMase D isoform exposures may contribute to the quantitative variation observed in the transcriptional responses. Whole venoms contain additional components that can modulate keratinocyte activation, whereas recombinant SMase D isoforms isolate the specific contribution of this toxin family, which may accentuate or attenuate particular pathway signatures depending on dose and context.

Remarkably, tetracycline reversed this signature by downregulating *IL1RL1* while restoring *SOX2* and *SOX18* expression, irrespective of the venom or toxin class. Restored *SOX2* and *SOX18* expression facilitates wound healing by inducing growth factors and extracellular matrix proteins that promote keratinocyte proliferation, migration, and angiogenesis, thereby coordinating epidermal and dermal repair and remodeling (Uchiyama et al., 2019; Darby et al., 2001; Grimm et al., 2020).

Regarding inflammation, tetracycline suppressed *IL1RL1*, a receptor that amplifies IL-1–mediated signaling in keratinocytes. As previously demonstrated, SMase D activates the IL-1 signaling cascade in keratinocytes, leading to exaggerated inflammatory responses (Pinto et al., 2023), and *IL1RL1* upregulation further contributes to this amplification. Thus, *IL1RL1* downregulation by tetracycline likely attenuates upstream IL-1-driven inflammation and limits neutrophil infiltration, a hallmark of expanding dermonecrotic lesions. These findings are consistent with *in vivo* studies showing reduced neutrophil recruitment in tetracycline-treated rabbit models of cutaneous loxoscelism (Paixao-Cavalcante et al., 2006; Ishikawa et al., 2009; Tambourgi et al., 2005), reinforcing the clinical relevance of these mechanisms.

One key molecular hallmark of cutaneous loxoscelism is the induction of matrix metalloproteinases (MMPs), particularly MMP-2, MMP-7, and MMP-9 (Tambourgi et al., 2005; Paixao-Cavalcante et al., 2006; Paixao-Cavalcante et al., 2007; Corrêa et al., 2016). These enzymes play a central role in degrading extracellular matrix (ECM) components, which not only contributes to the progression of dermonecrotic lesions but also facilitates inflammatory cell recruitment and perpetuates local inflammation. Moreover, chronic non-healing wounds are characterized by excessive and uncontrolled proteolysis, a process in which MMPs are key pathogenic mediators (Hanemaaijer et al., 1998; Caley, Martins, & O’Toole, 2015).

Corroborating previous studies from our group, in which we demonstrated that tetracycline reduces the secretion of MMP-2 and MMP-9 and prevents DNA degradation, ultimately preserving cell viability (Paixao-Cavalcante et al., 2006) and, abolishes the conversion of pro-MMP-7 into its active form and effectively inhibits *L. laeta* venom-induced dermonecrosis *in vivo* (Corrêa et al., 2016), our transcriptomic data show that tetracycline treatment downregulated the *MMP9* gene, which was upregulated by SMase D Class II treatment, as well as a reduced *MMP7* gene expression after its upregulation due to treatment with SMase D Class I.

In this context, tetracycline also sharply reversed the expression of other *MMP* genes that were increased by SMase D treatments but not by whole venoms, likely reflecting differences in exposure time or toxin concentration. Among these, *MMP3* is of particular interest, as it amplifies tissue destruction and inflammation by degrading extracellular matrix components such as collagen and fibronectin, thereby promoting dermal degradation, vascular breakdown, and ulcer formation (Mirastschijski et al., 2019).

Similarly, a slight downregulation of the *MMP10* gene was observed following *L. intermedia* venom treatment in the presence of tetracycline. MMP-10 acts as a context-dependent modulator of inflammation (McMahan et al., 2016), and in the setting of dermonecrosis, excessive or prolonged expression may compromise extracellular matrix integrity and hinder the healing process. Thus, even modest *MMP10* downregulation may reduce keratinocyte detachment and migration, and its expression suppression by tetracycline may help stabilize the wound edge, limiting excessive tissue remodeling and potentially favoring scar formation or a more controlled repair process (Caley et al., 2015). This broad-spectrum inhibition of tissue-degrading MMPs provides a mechanistic basis for the reduced dermonecrosis observed in tetracycline-treated preclinical models.

Interestingly, cells treated with Class I SMase D in the presence of tetracycline showed increased *MMP11* expression. Unlike tissue degrading MMPs, MMP-11 facilitates fibroblast migration, controlled collagen VI remodeling, and dermal matrix reorganization. These processes contributed to epithelial stability and wound closure, rather than matrix degradation (Krampert et al., 2004). Although tetracycline is generally associated with *MMP* downregulation, the specific upregulation of *MMP11* may reflect an adaptive ‘protective remodeling’ response following extensive extracellular matrix damage. In this context, a moderate increase in *MMP11* expression could indicate a compensatory mechanism that helps to preserve matrix integrity and promotes tissue repair (Stamenkovic, 2003; Caley et al., 2015).

Extending these findings, we observed that treatment of keratinocytes with *L. intermedia* venom in the presence of tetracycline led to a significant enrichment of IL-10 signaling, a pathway with well-established regulatory effects on inflammation and cutaneous wound healing (Short et al., 2023). Collectively, these observations highlight tetracycline as a potent MMP inhibitor capable of limiting venom/SMase D-induced proteolytic damage while simultaneously promoting a reparative microenvironment through *IL-10* upregulation, thereby fostering immune regulation and wound resolution.

Keratinocytes exposed to Class I and II SMase D isoforms display marked upregulation of inflammatory (*IL36G, IL1A, IL1B, IL1RL1, IL1RL2, IL1R2, CXCL1, CXCL8, IL6*) and proteolytic (*ADAM19*) genes, along with a wound-impairing signature characterized by *CCR10* downregulation and *ERRFI1* upregulation (Pinto et al., 2023). Balanced control of this inflammatory axis is critical: excessive activation causes collateral tissue damage, whereas insufficient activation predisposes to chronic, non-healing wounds (Ogawa, 2017; Soliman and Barreda, 2022; Gao et al., 2024). In this context, tetracycline largely reprogrammed the SMase D-induced profile, suppressing inflammatory and proteolytic mediators while restoring *CCR10* expression irrespective of the isoform, consistent with the known ability of tetracyclines to dampen IL-1/IL-6 signaling and MMP activity and thereby support tissue regeneration (Amin et al., 1996; Paixao-Cavalcante et al., 2007; Tambourgi et al., 2010; Peukert et al., 2021; Mehrabi et al., 2021).

Worthy of note, *CCR10* and its ligand *CCL27* are physiologically expressed in keratinocytes and are critical for the recruitment of epidermal precursor cells from the bone marrow to sites of injury, where they differentiate locally and accelerate wound closure (Inokuma et al., 2006; Fujimoto et al., 2008). Studies in *CCR10* ^*−/−*^ mice have demonstrated that loss of *CCR10* disrupts the immune homeostatic balance between effector and regulatory T cells in the skin, leading to exaggerated inflammatory responses and impaired resolution of cutaneous inflammation (Xia et al., 2014).

Despite the convergence of our datasets on inflammatory, proteolytic and reparative programs, several mechanistic aspects remain unresolved. In particular, the specific contribution of IL-1 family signaling, MMP activity and CCR10-dependent recruitment of epidermal precursors to dermonecrosis and subsequent wound healing is still incompletely defined. Our RNA-seq design and analysis follow current best practices and provide robust genome-wide insight, but we did not perform new functional assays in this study to validate each pathway individually. We therefore interpret the data in light of our previously published functional and histological work in the same model and present these axes as central, yet still testable, hypotheses that require targeted experimentation to clarify their roles in venom-induced pathology and tetracycline-mediated protection. Finally, because clinical management of loxoscelism remains debated, extrapolation of tetracycline’s effects from preclinical models to patients should be made cautiously, pending controlled clinical evaluation.

In summary, this study offers a transcriptomic and network-level view of how *L. laeta* and *L. intermedia* venoms and their SMase D Class I and II isoforms reprogram human keratinocytes and how tetracycline modulates these responses. Venoms and SMase D induced inflammatory and proteolytic gene expression profiles and downregulated programs related to epidermal morphogenesis, cell migration, and wound healing, whereas tetracycline co-treatment attenuated these changes and restored transcriptional programs associated with keratinocyte plasticity, SOX2/SOX18 expression, CCR10-related signaling, and tissue repair. Together with our previous functional data, these findings outline a mechanistic framework linking venom-induced dermonecrosis and impaired healing to specific transcriptional networks in keratinocytes and point to defined axes that now warrant targeted functional validation.

## Data Availability

The data have been deposited in the NCBI BioProject database with accession number PRJNA1448015.
